# Supplementary material for: Dementia Education and Training for the Multidisciplinary Student Healthcare Workforce: A Systematic Review
Source: Int J Geriatr Psychiatry. 2025 Jul 1;40(7):e70119. doi: 10.1002/gps.70119 (PMC12214054; doi:10.1002/gps.70119)
Supplement: Supplementary file 1 — Supporting Information S1 [file GPS-40-e70119-s001.docx]

**Table S1: Search strategy for PubMed (dates 2015- filter applied)**

| Terms |  |
| --- | --- |
| Education | (educat*[Title/Abstract] OR training[Title/Abstract] OR staff development[Title/Abstract] OR professional development[Title/Abstract] OR CPD[Title/Abstract] OR skills training[Title/Abstract] OR curricul*[Title/Abstract] OR learn*[Title/Abstract] OR teach*[Title/Abstract] OR workshop[Title/Abstract] OR module[Title/Abstract]) |
| Staff | ((professional[Title/Abstract] OR staff[Title/Abstract] OR worker[Title/Abstract] OR workforce[Title/Abstract] OR paid carer[Title/Abstract] OR aide[Title/Abstract] OR care worker[Title/Abstract] OR physician[Title/Abstract] OR doctor[Title/Abstract] OR student[Title/Abstract] OR nurse[Title/Abstract] OR therapist[Title/Abstract] OR social worker[Title/Abstract]) |
| Dementia | (dementia[Title/Abstract] OR "Frontotemporal dementia"[Title/Abstract] OR korsakof*[Title/Abstract] OR binswanger[Title/Abstract] OR "Progressive Supranuclear palsy"[Title/Abstract] OR alzheimer*[Title/Abstract] OR dement*[Title/Abstract] OR "Korsakoff Syndrome"[Title/Abstract] OR "Wernicke's Encephalopathy"[Title/Abstract] OR "Huntington's Disease"[Title/Abstract] OR "Multi Infarct"[Title/Abstract] OR "lewy bodies"[Title/Abstract] OR "Lewy Body Disease"[Title/Abstract] OR "kluver-bucy syndrome"[Title/Abstract] OR "Vascular dementia"[Title/Abstract] OR "Creutzfeldt-Jakob Syndrome"[Title/Abstract] OR "Alzheimer's Disease"[Title/Abstract])) |

**Table S2: Quality rating of studies for healthcare workers in training, using the MMAT**

|  | ***Qualitative*** | | | | | ***Quantitative non-randomised*** | | | | | ***Quantitative descriptive*** | | | | | ***Mixed Methods*** | | | | | |
| --- | --- | --- | --- | --- | --- | --- | --- | --- | --- | --- | --- | --- | --- | --- | --- | --- | --- | --- | --- | --- | --- |
|  | 1.1 | *1.2* | *1.3* | *1.4* | *1.5* | *3.1* | *3.2* | *3.3* | *3.4* | *3.5* | *4.1* | *4.2* | *4.3* | *4.4* | *4.5* | *5.1* | *5.2* | *5.3* | *5.4* | *5.5* |  |
| *Annear*^1^ | *x* | *x* | *x* | *x* | *x* | *x* | *x* | *x* | *-* | *x* |  |  |  |  |  | *x* | *x* | *x* | *x* | *x* |  |
| *Balzer*^2^ | *x* | *x* | *x* | *-* | *-* | *x* | *x* | *x* | *-* | *x* |  |  |  |  |  | *-* | *x* | *x* | *x* | *-* |  |
| *Banerjee*^3^ |  |  |  |  |  | *x* | *x* | *-* | *x* | *x* |  |  |  |  |  |  |  |  |  |  |  |
| *Bard* ^4^ |  |  |  |  |  | *x* | *-* | *x* | *-* | *x* |  |  |  |  |  |  |  |  |  |  |  |
| *Berning*^5^ |  |  |  |  |  | *x* | *x* | *x* | *-* | *x* |  |  |  |  |  |  |  |  |  |  |  |
| *Brown*^6^ | *x* | *x* | *x* | *x* | *x* | *x* | *x* | *-* | *-* | *x* |  |  |  |  |  | *-* | *x* | *x* | *x* | *-* |  |
| *Craig*^7^ |  |  |  |  |  | *x* | *x* | *x* | *-* | *-* |  |  |  |  |  |  |  |  |  |  |  |
| *Daley*^8^ | *x* | *x* | *x* | *x* | *x* |  |  |  |  |  |  |  |  |  |  |  |  |  |  |  |  |
| *Daley*^9^ |  |  |  |  |  | *x* | *x* | *-* | *x* | *-* |  |  |  |  |  |  |  |  |  |  |  |
| *Davison*^10^ | *x* | *x* | *x* | *x* | *x* |  |  |  |  |  | *x* | *-* | *x* | *-* | *-* | *-* | *x* | *x* | *x* | *-* |  |
| *DeCaro*^11^ |  |  |  |  |  | *x* | *x* | *-* | *-* | *x* |  |  |  |  |  |  |  |  |  |  |  |
| *Dellasega*^12^ | *x* | *x* | *x* | *x* | *x* |  |  |  |  |  |  |  |  |  |  |  |  |  |  |  |  |
| *Dingwall et al. (2017)* | *-* | *x* | *x* | *-* | *x* | *x* | *-* | *x* | *-* | *x* |  |  |  |  |  | *x* | *x* | *-* | *x* | *-* |  |
| *Dressel et al. (2023)* | *x* | *x* | *x* | *-* | *-* | *x* | *x* | *-* | *-* | *x* |  |  |  |  |  | *-* | *x* | *-* | *x* | *-* |  |
| *Frausing*^13^ | *x* | *x* | *x* | *x* | *x* |  |  |  |  |  |  |  |  |  |  |  |  |  |  |  |  |
| *Griffiths*^14^ |  |  |  |  |  | *x* | *x* | *-* | *-* | *x* |  |  |  |  |  |  |  |  |  |  |  |
| ***Grosvenor***^15^ | ***x*** | ***x*** | ***x*** | ***x*** | ***x*** |  |  |  |  |  |  |  |  |  |  |  |  |  |  |  |  |
| *Harrington*^11^ | *x* | *x* | *x* | *x* | *x* | *-* | *-* | *x* | *-* | *x* |  |  |  |  |  | *x* | *x* | *-* | *-* | *-* |  |
| *Hartung*^16^ |  |  |  |  |  | *x* | *x* | *-* | *-* | *x* |  |  |  |  |  |  |  |  |  |  |  |
| *Haugland*^17^ | *x* | *x* | *x* | *x* | *x* |  |  |  |  |  |  |  |  |  |  |  |  |  |  |  |  |
| *Jones*^18^ |  |  |  |  |  | *x* | *x* | *-* | *-* | *x* |  |  |  |  |  |  |  |  |  |  |  |
| *Long*^19^ |  |  |  |  |  | *-* | *x* | *x* | *-* | *-* |  |  |  |  |  |  |  |  |  |  |  |
| *Love*^20^ |  |  |  |  |  | *x* | *x* | *x* | *x* | *x* |  |  |  |  |  |  |  |  |  |  |  |
| *Mastel-Smith*^21^ | *x* | *x* | *x* | *x* | *x* | *-* | *x* | *x* | *-* | *x* |  |  |  |  |  | *x* | *x* | *x* | *x* | *-* |  |
| *Mosley*^22^ |  |  |  |  |  | *x* | *x* | *-* | *-* | *x* |  |  |  |  |  |  |  |  |  |  |  |
| *Naughton et al*^23^ | *x* | *x* | *x* | *x* | *x* |  |  |  |  |  |  |  |  |  |  |  |  |  |  |  |  |
| *Naughton*^24^ | *x* | *x* | *x* | *x* | *x* | *x* | *x* | *-* | *-* | *x* |  |  |  |  |  | *-* | *x* | *x* | *x* | *-* |  |
| *Niedens*^25^ | *x* | *x* | *x* | *x* | *x* | *x* | *x* | *-* | *-* | *x* |  |  |  |  |  | *x* | *x* | *x* | *x* | *-* |  |
| *Peng*^26^ | *x* | *x* | *x* | *-* | *x* | *x* | *x* | *x* | *-* | *-* |  |  |  |  |  | *-* | *x* | *x* | *x* | *-* |  |
| *Salajegheh* ^27^ | *x* | *x* | *x* | *x* | *x* |  |  |  |  |  |  |  |  |  |  |  |  |  |  |  |  |
| *Schwarz*^28^ |  |  |  |  |  | *-* | *x* | *x* | *-* | *x* |  |  |  |  |  |  |  |  |  |  |  |
| *Smyth*^29^ | *x* | *x* | *x* | *x* | *x* | *-* | *-* | *-* | *x* |  |  |  |  |  |  | *-* | *x* | *-* | *-* | *-* |  |
| *Um*^30^ | *x* | *x* | *x* | *x* | *x* |  |  |  |  |  |  |  |  |  |  |  |  |  |  |  |  |
| *Winter*^31^ | *-* | *x* | *-* | *x* | *-* |  |  |  |  |  |  |  |  |  |  |  |  |  |  |  |  |
| *Wonnacott*^32^ | *x* | *x* | *x* | *x* | *x* |  |  |  |  |  |  |  |  |  |  |  |  |  |  |  |  |

***‘X’*** = criteria met; **‘-‘** = criteria not met

**MMAT, 2018 methodological quality criteria:**

**1. Qualitative;** 1.1. Is the qualitative approach appropriate to answer the research question? 1.2. Are the qualitative data collection methods adequate to address the research question? 1.3. Are the findings adequately derived from the data? 1.4. Is the interpretation of results sufficiently substantiated by data? 1.5. Is there coherence between qualitative data sources, collection, analysis and interpretation?

**3. Quantitative non-randomised;** 3.1. Are the participants representative of the target population? 3.2. Are measurements appropriate regarding both the outcome and intervention (or exposure)? 3.3. Are there complete outcome data? 3.4. Are the confounders accounted for in the design and analysis? 3.5. During the study period, is the intervention administered (or exposure occurred) as intended?

**4. Quantitative descriptive;** 4.1. Is the sampling strategy relevant to address the research question? 4.2. Is the sample representative of the target population? 4.3. Are the measurements appropriate? 4.4. Is the risk of nonresponse bias low? 4.5. Is the statistical analysis appropriate to answer the research question?

**5. Mixed Methods;** 5.1. Is there an adequate rationale for using a mixed methods design to address the research question? 5.2. Are the different components of the study effectively integrated to answer the research question? 5.3. Are the outputs of the integration of qualitative and quantitative components adequately interpreted? 5.4. Are divergences and inconsistencies between quantitative and qualitative results adequately addressed? 5.5. Do the different components of the study adhere to the quality criteria of each tradition of the methods involved? Note: 5.5 was rated ‘Yes’ if individual Qualitative and Quantitative components were rated 4+.

**Table S3: Characteristics of studies rated <4 on the MMAT appraisal tool**

| **EXPERIENTIAL INTERVENTIONS INVOLVING VISITS OR SHARED ACTIVITIES WITH PEOPLE WITH DEMENTIA (N=3)** | | | | | | | | | | | | | |
| --- | --- | --- | --- | --- | --- | --- | --- | --- | --- | --- | --- | --- | --- |
| **Study**  **Country** | **Settings and Participants** | **Intervention** | **n** | **Control** | **n** | **n (%) with primary outcome)** | **Outcome (from baseline)** | **Outcomes: Kirkpatrick levels** | | | | **Study type** | **Validity score** |
|  |  |  |  |  |  |  |  | **1** | **2** | **3** | **4** |  |  |
| Daley^33^  UK | Healthcare students from 5 universities | TFD Programme: students visit a PLWD and their family carer (See Table 1) | 2700 | UT | 863 | 3312 (93%) | 24 months |  | ☺ |  |  | Quant | 3/5 |
| Mosley^22^  USA | Pharmacy students from 1 university | 3 outreach sessions where the students were paired with a PLWD to encourage fidget blanket use through patient-specific directions and demonstration. | 26 | _ | _ | 12/26 (46%) | PI^e^ |  | ☺ |  |  | Quant | 3/5 |
| Balzer^2^ Germany | Nursing and medical students | Lectures, problem-based learning tutorials and visits to care facilities. Contact hours with lecturers, tutors or healthcare representatives around 30 hours. | 18 | _ | _ | 18/18 (100%) | PI | ☺ | ☺ |  |  | MM | 3/5 |
| **SKILLS TRAINING DURING CLINICAL PLACEMENT (N=3)** | | | | | | | | | | | | | |
| Hartung^16^  Canada | 1^st^ year nursing students completing clinical placements at geriatric healthcare settings | 2.75-hour workshop that applied a person-centred communication framework when caring for PLWD experiencing responsive behaviours during the students' 13-week clinical placements. | 43 | - | - | 33/43 (76.7%) | PI and 10-week follow-up |  |  |  | ☺ | Quant | 3/5 |
| Naughton^23^ UK | Pre-registration student nurses in 5 older adult units across two hospitals | 2.5-hour training sessions using VERA framework communication tool, plus reflective discussions, in placement | 51 | UT | 66 | 52/117 (44%) | PI | ☺ | ☺ |  |  | MM | 3/5 |
| Smyth^29^  Ireland | 2^nd^ year undergraduate nursing students in a residential facility | 2.5-hour, in-person VERA communication skills training by two trained researchers, strategies used during placement. | 10 | UT | 6 | 6/6 (100%) | PI |  | ☺ |  |  | MM | 1/5 |
| **SELF-DIRECTED ONLINE LEARNING (N=3)** | | | | | | | | | | | | | |
| Brown^6^ USA | Pre-clinical nursing students from 3 nursing schools in Connecticut and Hawaii | Modular, flipped classroom curriculum showing character animation techniques and videos teaching cognition and dementia, respectively (2 sessions). | 223 | - | - | 152/223 (68%) | PI | ☺ | ☺ |  |  | MM | 3/5 |
| Craig^7^  Northern Ireland | 1^st^ year undergraduate nursing students | Digital game with multiple-choice questions about dementia. Game about 90 seconds, can be played multiple times. | 452 | - | - | 334 (73.9%) | PI |  | ☺ |  |  | Quant | 3/5 |
| Long^19^  USA | Nursing students from Lamar University | Four online, interactive educational modules, focused on caring for PLWD, clinical reasoning abilities and student confidence through video vignettes. | 65 | - | - | 65/65 (100%) | PI |  | ☺ |  |  | Quant | 2/5 |
| **DEMENTIA AWARENESS (DEMENTIA FRIENDS) TRAINING (N=2)** | | | | | | | | | | | | | |
| Griffiths^14^  Malaysia | Pharmacy and medical undergraduate students from 1 University | One hour dementia education; dispels myths around dementia; demonstration of content, interactive activities in 5 teams, videos, discussion, case study | 112 | - | - | 97/112 (86.6%) | PI |  | ☺ |  |  | Quant | 3/5 |
| Davison^10^ UK | First-year healthcare students (medicine, nursing, speech and language therapy, OP, physiotherapy, pharmacy, paramedic science) from University of East Anglia | Inter-professional team created a learning package for students to gain a Tier 1 DA^h^ qualification, using the platform of an existing inter professional learning module. It comprised of 1-hour DF^i^ session (face-to-face or online), a reflective workbook and 10 multiple choice questions whereby 8/10 was required for the DA qualification. | 60 | - | - | 57/60 (95%) | PI | ☺ | ☺ |  |  | MM | 3/5 |
| **EXPERIENCING AND REFLECTING ON SIMULATION/FICTIONAL SCENARIOS (N=6)** | | | | | | | | | | | | | |
| Harrington^11^  USA | Nurse practitioner students from one university and one nursing college | VDT^j^: Trained facilitators guide students through an eight-minute experience of a PLWD perspective, as they try to perform five daily activities, followed by a 45-minute focus group. | 44 | - | - | 20/44 (45.5%) | PI |  | ☺ |  |  | MM | 2/5 |
| Schwarz^28^  USA | 1^st^ year DPT^k^ students at a local memory care facility | 2-hour experimental learning activity involving a brief, simulated dementia experience, educational session, observational facility tour and a debriefing session. Facilitated by dementia-training educators | 82 | - | - | 80/82 (95.4%) | PI and nine-month follow-up |  | ☺ |  |  | Quant | 3/5 |
| Dingwall^34^ UK | 3^rd^ year nursing and social work students from one university | ‘Sliding Doors’, drama-based, educational one-day workshop; dramatized scenarios to stimulate discussions. | 63 | - | - | Unclear | PI | ☺ | ☺ |  |  | MM | 3/5 |
| Bard^4^  USA | 2^nd^ year medical students | 30-minute VR simulation session, through the perspective of a PLWD. | 149 | - | - | 149/149 (100%) | PI |  | ☺ |  |  | Quant | 3/5 |
| Peng^26^  China | 2^nd^ year undergraduate nursing students | Movie overview of ‘Still Alice’, about a PLWD; 8-minute, 5-task modified VDT. | 45 | - | - | N/A | PI | ☺ | ☺ | ☺ |  | MM | 3/5 |
| Winter^31^  UK | All 3^rd^ year medical students from one university | 12 simulation days over 9 months, based on communicating with a person with dementia in several scenarios facilitated by doctors, nurses and occupational therapists specialised in dementia care. Students managed simulated scenarios in pairs, followed by a 30-minute debrief. | 145 | - | - | N/A | PI | ☺ | ☺ |  |  | Qual^m^ | 2/5 |
| **CLASSROOM-BASED LEARNING (N=3)** | | | | | | | | | | | | | |
| Jones^18^  UK | Student paramedics from 1 university | A 6-hour education program with didactic teaching, collaborative and reflective learning. Face to face by specialist dementia educator; involved quizzes, case studies, videos; prerequisite learning | 43 | - | - | 32/43 (74.4%) | PI | ☺ | ☺ |  |  | Quant | 3/5 |
| DeCaro^35^ USA | Medical students, undergraduate and graduate students interested in medicine | 6 educational and 3 outreach events; monthly dementia focused didactic meetings and outreach focusing on Black participant recruitment | 37 | _ | _ | 20/37 (54%) | PI |  | ☺ |  |  | Quant | 3/5 |
| Dressel ^36^et al. (2023)  Germany | Nutrition therapy and counselling, speech language pathology and physiotherapy students from 1 University | Interprofessional and competency-based education; three-day workshop focusing on patient-centred care and inter professional collaboration; case-based leaning in simulated interprofessional case-conferences and peer teaching. | 42 | - | - | 42/53 (79.2%) | PI |  | ☺ |  |  | MM | 2/5 |

**Legend**: DA: Dementia Awareness; DF: Dementia Friends; DPT: Doctor of Physical Therapy; MM: mixed methods; PI: post-intervention; PLWD: person living with dementia; Qual: qualitative; Quant: quantitative; TFD: Time for Dementia; UT: Usual Training; VERA: Validation, Emotion, Reassurance, Activity; VDT: Virtual Dementia Tour; VR: Virtual Reality;

References

1. Annear MJ, Lea E, Lo A, Tierney L, Robinson A. Encountering aged care: a mixed methods investigation of medical students’ clinical placement experiences. *BMC Geriatr*. 2016;16:38. doi:10.1186/s12877-016-0211-8

2. Balzer K, Schröder R, Junghans A, Stahl U, Träder JM, Köpke S. Improving competencies in evidence-based dementia care: Results from a pilot study on a novel inter-professional training course (the KOMPIDEM project). *GMS J Med Educ*. 2016;33(2):Doc35. doi:10.3205/zma001034

3. Banerjee S, Jones C, Wright J, et al. A comparative study of the effect of the Time for Dementia programme on medical students. *Int J Geriatr Psychiatry*. 2021;36(7):1011. doi:10.1002/gps.5532

4. Bard JT, Chung HK, Shaia JK, Wellman LL, Elzie CA. Increased medical student understanding of dementia through virtual embodiment. *Gerontol Geriatr Educ*. 2023;44(2):211-222. doi:10.1080/02701960.2022.2067850

5. Berning MJ, Parkinson A, Tessier KM, Pejsa L, McCarthy TC, Ratner ER. Effect of a dementia friends information session on health professional students’ attitudes and knowledge related to dementia. *Gerontol Geriatr Educ*. 2023;44(2):185-195. doi:10.1080/02701960.2022.2123319

6. Brown B, Kang G, Schwartz A, et al. Cognition and dementia with Raymond and Brain: Curriculum development and evaluation using interactive animated flipped-classroom modules to impact nursing students’ attitude toward dementia care. *Nurse Educ Pract*. 2023;71:103696. doi:10.1016/j.nepr.2023.103696

7. Craig S, Stark P, Wilson CB, Carter G, Clarke S, Mitchell G. Evaluation of a dementia awareness game for undergraduate nursing students in Northern Ireland: a Pre-/Post-Test study. *BMC Nurs*. 2023;22(1):177. doi:10.1186/s12912-023-01345-2

8. Daley S, Feeney Y, Grosvenor W, et al. A qualitative evaluation of the effect of a longitudinal dementia education programme on healthcare student knowledge and attitudes. *Age Ageing*. 2020;49. doi:10.1093/ageing/afaa182

9. Daley S, Hebditch M, Jones C, et al. Time for Dementia: Quantitative evaluation of a dementia education programme for healthcare students. *Int J Geriatr Psychiatry*. 2023;38(5):e5922. doi:10.1002/gps.5922

10. Davison E, Housden S, Lindqvist S. Using interprofessional dementia learning opportunities to prepare the future healthcare workforce: findings from a pilot study. *J Interprof Care*. 2019;33(6):816-819. doi:10.1080/13561820.2018.1551863

11. Harrington CC, Neil JA, Hardin SR, Roberson DW. Is Perception Reality? Using Person-in-Context Simulation to Promote Empathic Understanding of Dementia Among Nurse Practitioner Students. *Nurs Educ Perspect*. 2021;42(6):377-379. doi:10.1097/01.NEP.0000000000000780

12. Dellasega C, George DR, Lokon E. The transformative power of participating in Opening Minds Through Art (OMA), an expressive arts program for medical students. *Gerontol Geriatr Educ*. 2023;0(0):1-13. doi:10.1080/02701960.2023.2255537

13. Frausing KP, Stamp AS. Making a difference: Students’ experiences with a dementia care program. *Gerontol Geriatr Educ*. 2021;42(1):126-139. doi:10.1080/02701960.2019.1659256

14. Griffiths AW, Cheong WL, Saw PS, Parveen S. Perceptions and attitudes towards dementia among university students in Malaysia. *BMC Med Educ*. 2020;20(1):82. doi:10.1186/s12909-020-1972-5

15. Grosvenor W, Gallagher A, Banerjee S. Reframing dementia: Nursing students’ relational learning with rather than about people with dementia. A constructivist grounded theory study. *Int J Geriatr Psychiatry*. 2021;36(4):558-565. doi:10.1002/gps.5452

16. Hartung B, Freeman C, Grosbein H, Santiago AT, Gardner S, Akuamoah-Boateng M. Responding to responsive behaviours: A clinical placement workshop for nursing students. *Nurse Educ Pract*. 2020;45:102759. doi:10.1016/j.nepr.2020.102759

17. Haugland VL, Reime MH. Scenario-based simulation training as a method to increase nursing students’ competence in demanding situations in dementia care. A mixed method study. *Nurse Educ Pract*. 2018;33:164-171. doi:10.1016/j.nepr.2018.08.008

18. Jones D, Capstick A, Faisal M, Frankland J. The impact of dementia education on student paramedics’ preparedness to care, knowledge, confidence and attitudes towards dementia: an analytic survey. *Br Paramed J*. 2023;8(1):9-17. doi:10.29045/14784726.2023.6.8.1.9

19. Long EM, Hale RL. Improving nursing students’ confidence in caring for persons with dementia. *Geriatr Nurs N Y N*. 2022;43:309-311. doi:10.1016/j.gerinurse.2021.04.017

20. Love T, Wiese LAK, Duncan V, Bertrand H. Does self-directed learning address gaps in nursing student knowledge of Alzheimer’s disease? *Educ Gerontol*. 2023;49(8):673-686. doi:10.1080/03601277.2022.2148445

21. Mastel-Smith B, Kimzey M, Garner J, Shoair OA, Stocks E, Wallace T. Dementia care boot camp: interprofessional education for healthcare students. *J Interprof Care*. 2020;34(6):799-811. doi:10.1080/13561820.2019.1696287

22. Mosley B, Kroustos KR, Sobota KF, Brooks R. Enhancing student-pharmacists’ professional development through community outreach with dementia population. *Ment Health Clin*. 2020;10(1):6-11. doi:10.9740/mhc.2020.01.006

23. Naughton C, Beard C, Tzouvara V, et al. A dementia communication training intervention based on the VERA framework for pre-registration nurses: Part I developing and testing an implementation strategy. *Nurse Educ Today*. 2018;63:94-100. doi:10.1016/j.nedt.2018.01.023

24. Naughton C, Beard C, Tzouvara V, et al. A feasibility study of dementia communication training based on the VERA framework for pre-registration nurses: Part II impact on student experience. *Nurse Educ Today*. 2018;63:87-93. doi:10.1016/j.nedt.2018.01.024

25. Niedens M, Yeager A, Vidoni ED, et al. A Collaborative Approach to Dementia Inclusion in Social Work Education: The Dementia Intensive. *J Soc Work Educ*. 2022;59(2):493. doi:10.1080/10437797.2022.2039820

26. Peng X, Wu L, Xie X, Dai M, Wang D. Impact of Virtual Dementia Tour on empathy level of nursing students: A quasi-experimental study. *Int J Nurs Sci*. 2020;7(3):258-261. doi:10.1016/j.ijnss.2020.06.010

27. Salajegheh M, Sohrabpour AA, Mohammadi E. Exploring medical students’ perceptions of empathy after cinemeducation based on Vygotsky’s theory. *BMC Med Educ*. 2024;24(1):94. doi:10.1186/s12909-024-05084-z

28. Schwarz B, Richardson MV, Camp K, Thomas R. Comparison of face to face and online delivery of a dementia-specific experiential learning activity. *Gerontol Geriatr Educ*. Published online June 21, 2024:1-10. doi:10.1080/02701960.2024.2366279

29. Smyth S, Dempsey L, Jordan F, et al. Perceptions and experiences of nursing students communicating with people living with dementia: The validation, emotion, reassure, activity (VERA) communication skills framework. *Int J Older People Nurs*. 2023;18(3):e12537. doi:10.1111/opn.12537

30. Um YJ. Nursing students’ simulated home-visit learning experiences with dementia -a qualitative research. *BMC Nurs*. 2023;22(1):70. doi:10.1186/s12912-023-01232-w

31. Winter R, Al-Jawad M, Harris R, Wright J. Learning to communicate with people with dementia: Exploring the impact of a simulation session for medical students (Innovative practice). *Dement Lond Engl*. 2020;19(8):2919-2927. doi:10.1177/1471301219845792

32. Wonnacott L, Banerjee S, Hicks B, Daley S. Understanding the experience of time for dementia education programme on undergraduate radiography students. *Radiography*. 2023;29:S46-S51. doi:10.1016/j.radi.2023.02.020

33. Daley S, Hebditch M, Jones C, et al. Time for Dementia: Quantitative evaluation of a dementia education programme for healthcare students. *Int J Geriatr Psychiatry*. 2023;38(5):e5922. doi:10.1002/gps.5922

34. Dingwall L, Fenton J, Kelly TB, Lee J. Sliding doors: Did drama-based inter-professional education improve the tensions round person-centred nursing and social care delivery for people with dementia: A mixed method exploratory study. *Nurse Educ Today*. 2017;51:1-7. doi:10.1016/j.nedt.2016.12.008

35. DeCaro R, O’Connor MK, DiTerlizzi C, Sekyi-Appiah N, Polk J, Budson AE. Educating students while recruiting underrepresented populations for Alzheimer’s disease research: the Student Ambassador Program. *BMC Med Educ*. 2022;22(1):707. doi:10.1186/s12909-022-03749-1

36. Dressel K, Ablinger I, Lauer AA, et al. Interprofessional education: a necessity in Alzheimer’s dementia care-a pilot study. *Front Med*. 2023;10:1235642. doi:10.3389/fmed.2023.1235642
